# Experimental and Computational Simulation of the Prebiotic Peptide Bond Formation Driven by Wet-Dry Cycles and Gamma Ionizing Radiation: An Insight into Molecular Evolution

**DOI:** 10.1007/s00239-026-10309-4

**Published:** 2026-04-06

**Authors:** E. Fonseca-González, C. A. Fuentes-Carreon, A. Heredia-Barbero

**Affiliations:** 1 Laboratorio de Evolución Química, Departamento de Química de Radiaciones y Radioquímica, Instituto de Ciencias Nucleares, Circuito, Exterior s/n, Ciudad Universitaria, Col. Universidad Nacional Autónoma de México, Alc. Coyoacán, Apartado Postal 70-543, Ciudad de México, C.P. 04510 México; 2 Network of Researchers on the Chemical Emergence of Life, NoRCEL Institute Leeds, Leeds, LS7 3RB UK; 3 Facultad de Química, Universidad Nacional Autónoma de México, Ciudad Universitaria, CDMX, Ciudad de México, 04510 México

**Keywords:** Prebiotic chemistry, Peptide bond formation, Gamma radiation, Wet-dry cycles, Molecular evolution, Amide I and Amide II infrared bands, Infrared spectroscopy, Differential scanning calorimetry, Thermal gravimetric analysis

## Abstract

The origin of life through prebiotic chemistry and molecular evolution processes is a significant and unresolved issue in science. A key part of this process is how simple molecules, such as amino acids, transition into functional polymers, like peptides, which can store information and facilitate reactions. During this transition, wet-dry cycles and the interaction with ionizing radiation are essential mechanisms for increasing molecular complexity. This process involves the repeated hydration and dehydration of organic compounds, promoting the formation of peptide bonds that connect amino acids into longer chains. On a faster-spinning ancient Earth, these cycles might have been more relevant for the shorter days. In this scenario, a faster rotation rate affected the climate, tidal forces, and evaporation rates. The transition from simple amino acids to functional peptides remains a central question in understanding the complexation of molecules that led to life’s origins. This work investigates how hydration-dehydration cycles impact solid-state gamma irradiated (40 kGy) DL-glutamic acid oligomerization. Infrared spectroscopy analysis confirms the presence of the characteristic amide I and II bands (approximately 1700 –1500 cm⁻¹), indicating an apparent change in peptide bond formation in gamma-irradiated samples. DSC and TGA thermal analysis reveal a contrasting difference in the thermograms from the control and gamma-irradiated samples. Thermal analyses demonstrate enhanced thermal stability in irradiated samples, agreeing with HyperChem computer simulations. The increase in stability in the oligomerization process. These findings support the model where cosmic ionizing radiation synergized with more frequent wet-dry cycles to promote prebiotic peptide synthesis. Our preliminary results substantiate the possibility that dry-wet cycles and ionizing irradiation on ancient Earth might have had planetary conditions to drive the molecular evolution towards abiotic synthesis of peptides.

## Introduction

The origin of life remains one of the most profound and unresolved questions in science (Pascal et al. 2006; Cleaves et al. 2014; Butch et al. 2021; Malaterre et al. 2022; Sydow et al. 2022; Das 2022; Zheng 2023). A critical step in this process was the transition from simple prebiotic molecules to functional polymers, such as peptides and nucleic acids, capable of storing information and catalyzing reactions (Mosqueira et al. 2008; Yang et al. 2025). Under this topic, it is relevant to understand the role of amino acids under prebiotic conditions and the possible way that they could self-assemble to form new structures and catalytic activities (Bar-Nun et al. 1994).

Wet-dry cycles are a compelling mechanism for driving molecular complexity (Fox and Harada 1960; Mamajanov et al. 2014; Forsythe et al. 2015; Song et al. 2024) among the various environmental factors that could have facilitated this transition. These cycles, which involve repeated hydration and dehydration of organic compounds, have promoted the formation of peptide bonds, enabling the oligomerization of amino acids into longer chains with increasingly sophisticated properties (Rodriguez-Garcia et al. 2015; Canavelli et al. 2019; Campbell et al. 2019).

In parallel, ionizing radiation represents another fundamental environmental factor that could have contributed to molecular evolution on the early Earth. It is essential to recognize that the radiative environment of the early Earth was multifaceted and dynamic. Beyond the gamma radiation arising from the decay of terrestrial radioisotopes, sources such as galactic cosmic rays (GCRs) and solar energetic particles (SEPs) contributed significantly to chemical processing during the Hadean and early Archean eons (Airapetian et al. 2016). GCRs, composed of high-energy particles capable of deep atmospheric penetration, and SEPs, whose fluxes were substantially more intense due to the heightened activity of the young Sun, would have driven radiolysis and free radical generation in aqueous media (Kobayashi et al. 1998; Rimmer and Helling 2016). The radiative landscape of the early Earth was dominated by high-energy sources significantly more intense than those of today. Primary sources of ionizing radiation included the decay of long-lived radionuclides (^40^K, ^232^Th, ^235,238^U) and short-lived isotopes (e.g., ^26^Al), which provided a dose rate approximately 3 to 5 times higher during the Hadean eon (Karam and Leslie 2005). In our experimental model, the use of gamma radiation serves as a proxy for the total ionizing dose and the molecular transformation mechanisms driven by this diverse radiative spectrum, which together facilitated the chemical complexity required for peptide bond formation under hydration-dehydration cycles. By generating highly reactive radical species through water radiolysis and direct interactions with organic molecules, γ-irradiation may have simultaneously induced fragmentation and promoted recombination, thereby expanding the range of chemical pathways available for prebiotic polymerization (Ershov 2022; Rodriguez et al. 2024).

In the study reported by (Kunikane and Sugai 1971) L- and DL-polyglutamic acids were irradiated using a Co-60 source. Upon irradiation, the formation of free radicals was detected, predominantly associated with the alpha carbon. The authors noted that DL-polyglutamic acid exhibited greater susceptibility to destabilization under ionizing radiation. Additionally, other investigations have indicated that radiolysis primarily results in the oxidative decarboxylation of the side-chain carboxyl group, leading to the formation of an aldehyde moiety at the carbon adjacent to the original carboxyl group. This structural modification corresponds to a characteristic mass decrease of approximately 30 Da (Xu and Chance 2004). In this prebiotic context, four billion years ago, during the Hadean era, the Earth’s geologic history and spinning rate differed significantly from today (Peltier 2007) in the average day length on Earth that could last less than 10 h (Arbab 2009). The Earth’s faster rotation during the Hadean and early Archean eons would have created pronounced diurnal cycles, with significant implications for prebiotic chemistry. This accelerated rotation generated more frequent temperature fluctuations between day and night, particularly in shallow aquatic environments such as tidal pools, hydrothermal fields, and evaporative basins (Ross and Deamer 2016). These dynamic systems would have experienced repeated wet-dry cycles that may have served as natural reactors for molecular evolution (Mamajanov et al. 2014). The cyclical nature of these environments facilitated the concentration of organic compounds and the removal of reaction byproducts (including water from condensation reactions), namely, it promoted the formation of increasingly complex polymers through continuous dehydration-rehydration processes (Saladino et al. 2019; Campbell et al. 2019). In addition, the thermodynamic challenge of peptide bond formation in aqueous environments presents a fundamental paradox for origins-of-life scenarios (Ross and Deamer 2016; Vitas and Dobovišek 2025). Because condensation reactions between amino acids are energetically unfavorable in bulk water due to competing hydrolysis, specific environmental conditions might overcome this limitation. For instance, mineral surfaces, particularly those of clays, sulfides, and silica, exhibit remarkable chemical pathways that enhance the catalytic properties for peptide synthesis through multiple mechanisms (Porter et al. 1998; Ferris 2006; Lambert 2008; Georgelin et al. 2013). Under dehydrating conditions, the thermodynamic landscape shifts dramatically in favor of condensation reactions. Moreover, experimental studies have revealed that amino acid crystals can facilitate amide bond formation when subjected to thermal fluctuations (Cervantes De La Cruz et al. 2017). Furthermore, prior to the formation of the ozone layer, intense solar UV radiation and the potential formation of UV-induced peroxides would have been severe destructive agents for organic molecules, similar to current Martian conditions (Cockell 2000). However, ionizing radiation like gamma rays possesses high penetration depth, allowing for chemical processing in sheltered niches—such as mineral pores or sediment layers—where molecules would be protected from direct UV photolysis. In these microenvironments, the synergy between wet-dry cycles and ionizing radiation could still favor polymerization despite the harsh surface environment. The crystalline matrix provides structural organization and protection from bulk water, while thermal gradients drive continuous dissolution and recrystallization cycles, promoting the formation of oligomers. As in other solids, gamma irradiation may reduce certain crystal unit cell parameters, thereby promoting self-assembly and structural organization, and increasing the diversity in possible prebiotic molecular evolution processes through lattice defect formation (El-Saady et al. 2023). Furthermore, the evolutionary trajectory from simple oligomers to functional polymers under these conditions appears to follow non-random pathways (Fox and Harada 1960; Küppers and Woolley 1983; Fox 1995), conceivably exhibiting primitive catalytic capabilities over amino acids in the water solution. Other authors have already suggested similar phenomena (Bar-Nun et al. 1994; Lange et al. 2020; Hlouchová 2024). In this work, we selected glutamic acid due to its documented presence in carbonaceous meteorites and its intrinsic catalytic properties (Hubert et al. 1963; Abbasi and Hatamjafari 2013; Farhad 2016; Khandan-Barani et al. 2017). We propose that its gamma-irradiated form is a plausible prebiotic agent for driving peptide bond formation, thereby increasing structural and physicochemical diversity to promote molecular evolution processes.

## Materials and methods

**Fig. 1:**
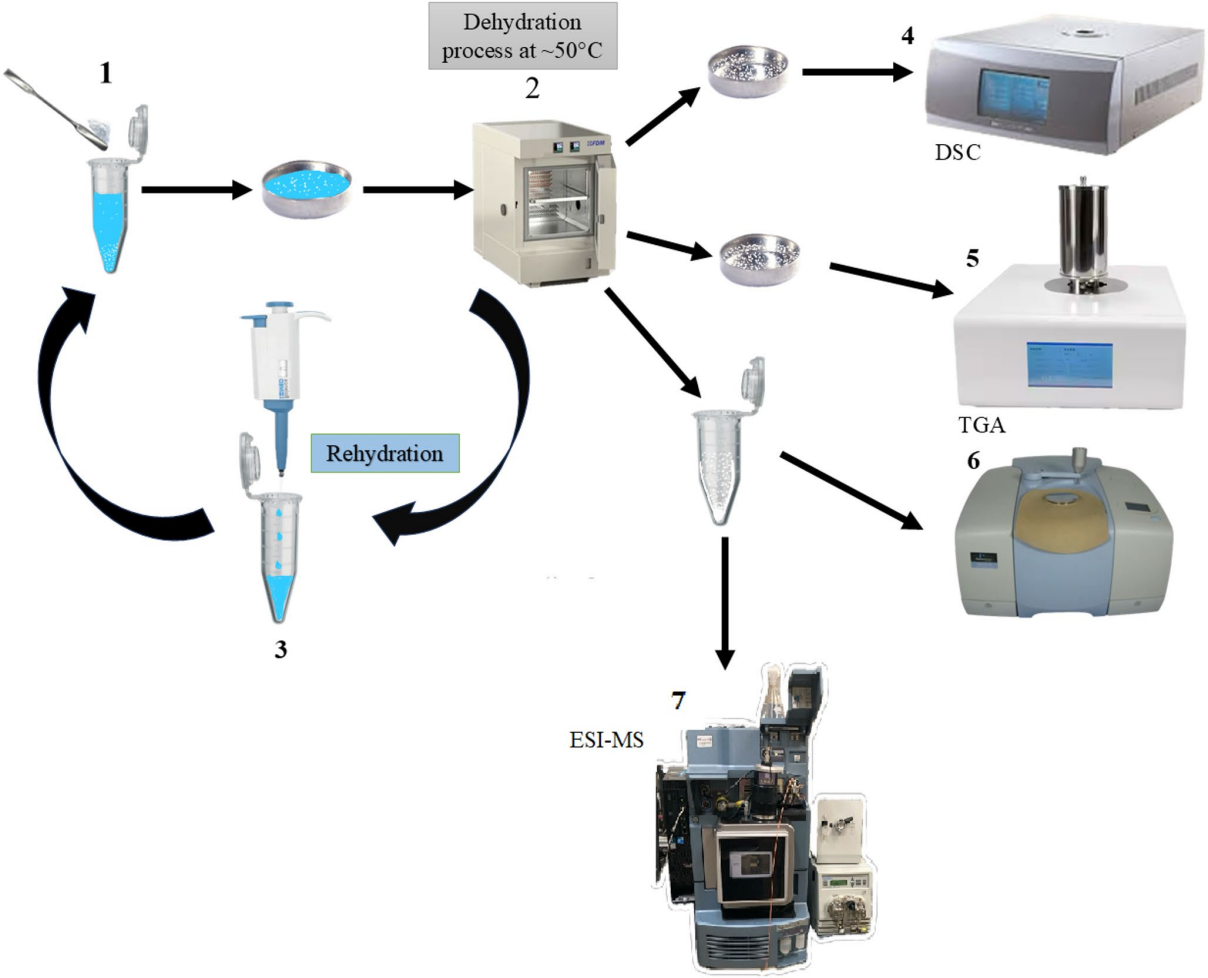
Schematic of the experimental procedure. Samples were derived from aqueous DL-glutamic acid solutions, followed by dehydration in a furnace at 50 °C. They underwent sequential rehydration to simulate hydration-dehydration cycles and were analyzed using Fourier Transform Infrared Spectroscopy (FTIR), Differential Scanning Calorimetry (DSC), Thermogravimetric Analysis (TGA), and Mass spectrometry. Flowchart of the DL-glutamic acid experiment in aqueous solution

Samples were prepared using DL-glutamic acid powder (100 mg per sample; Sigma-Aldrich, 98% HPLC CAS-No. 19285-83-7). Prior to any aqueous processing, the solid-state monomer was irradiated at a dose of 40 kGy using a ^60^Co source at the Instituto de Ciencias Nucleares, Universidad Nacional Autónoma de México. The radioactive material is stored in a 5.2 m deep pool, shielded with concrete and a stainless steel jacket(Cruz 1997; Barragán Mayet et al. 2023). This dose was selected based on the estimates by (Karam and Leslie 2005), where an exposure of ~ 0.5 mGy per year during the Hadean eon implies that our 40 kGy simulation corresponds to approximately 80 million years of cumulative exposure. Following irradiation, aqueous solutions were prepared by dissolving both gamma-irradiated and non-irradiated powder in 0.2 mL of Milli-Q water (filtered through a Millipak Gamma Gold 0.22 μm filter, Merck KGaA). These solutions were placed in Eppendorf tubes (Fig. 1) with a concentration carefully chosen near the solubility limit to maximize molecular proximity during the subsequent steps. The samples were then subjected to 5 wet-dry cycles. Each cycle consisted of a 48-hour period in a laboratory oven at a constant temperature of 50 °C until complete dehydration was achieved. The water used had a pH below 7.0, although atmospheric CO_2_ likely influenced this value during the open-vial thermal processing.

### Optical Microscopy

**Fig. 2:**
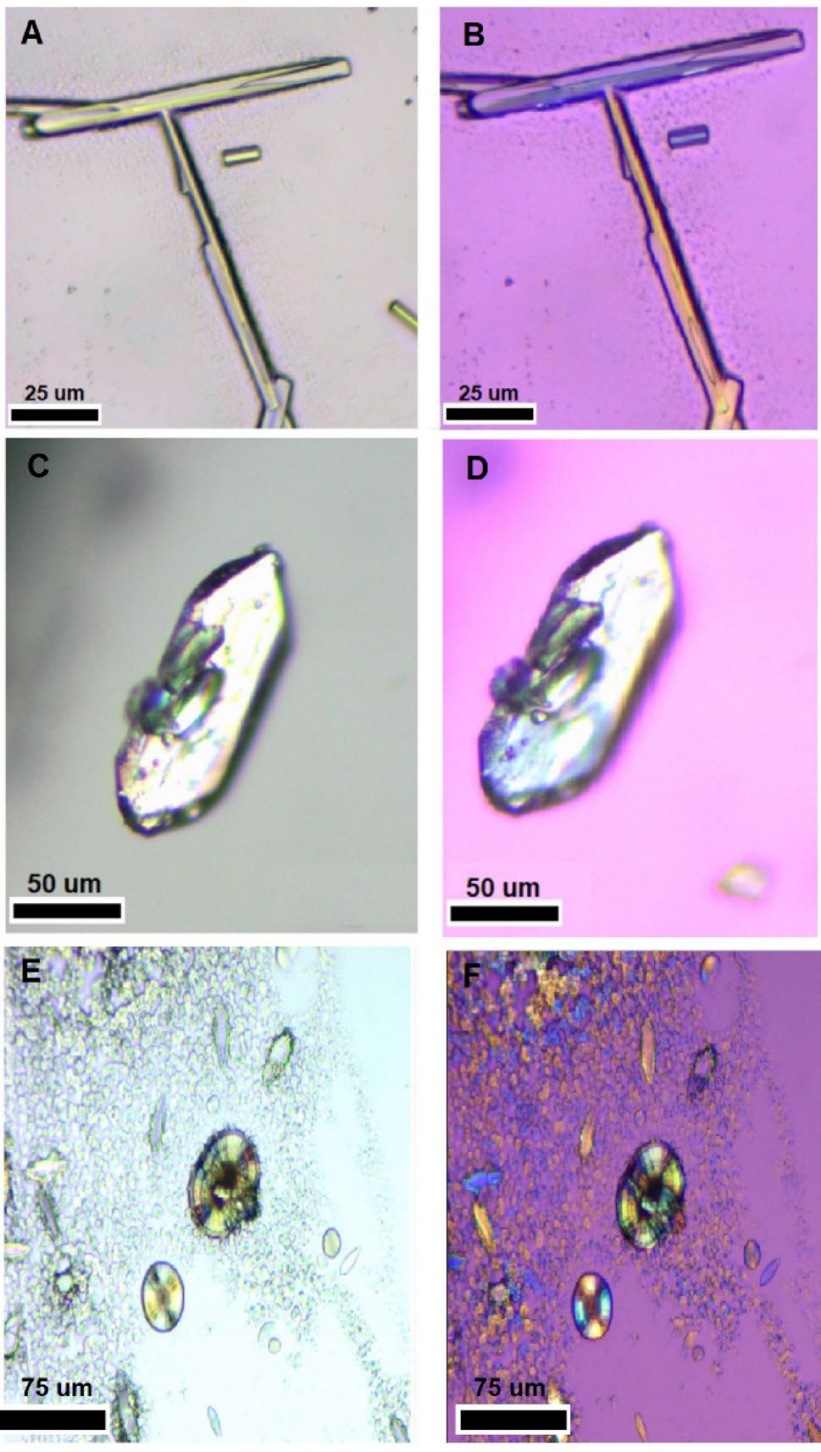
Unpolarized light (left: **A**, **C**, **E**) and polarized light micrographs (right: B, D, F) of DL-glutamic acid crystals after wet-dry cycle. **A**–**D** Control samples (non-irradiated) showing the coexistence of different crystalline habits produced by the cycling process: tubular crystals (**A**, **B**) and rice-like structures (**C**, **D**). **E**, **F** Pre-irradiated samples (40 kGy) exhibiting distinct, predominantly spherical morphologies with complex internal architectures. All crystals displayed optical polarization activity (right column), confirming their anisotropic properties. The transition to spherical shapes in the irradiated group suggests that radical-induced nucleation, coupled with the cycling process, significantly alters the recrystallization pathway compared to the control

The crystals obtained from pristine non-irradiated controls (untreated monomer powder) and after gamma irradiation were cast in glass microscope slides. Images of unpolarized and polarized light were taken using a Smart-POL polarizing microscope (Drawell, Chongqing, China) (Fig. 2). A polarized light microscope is essential for studying organic molecules, as it reveals birefringence and crystal structure. It also identifies molecular orientation and polymorphism, providing valuable insights into molecular interactions.

### Mass Spectrometry

**Fig. 3:**
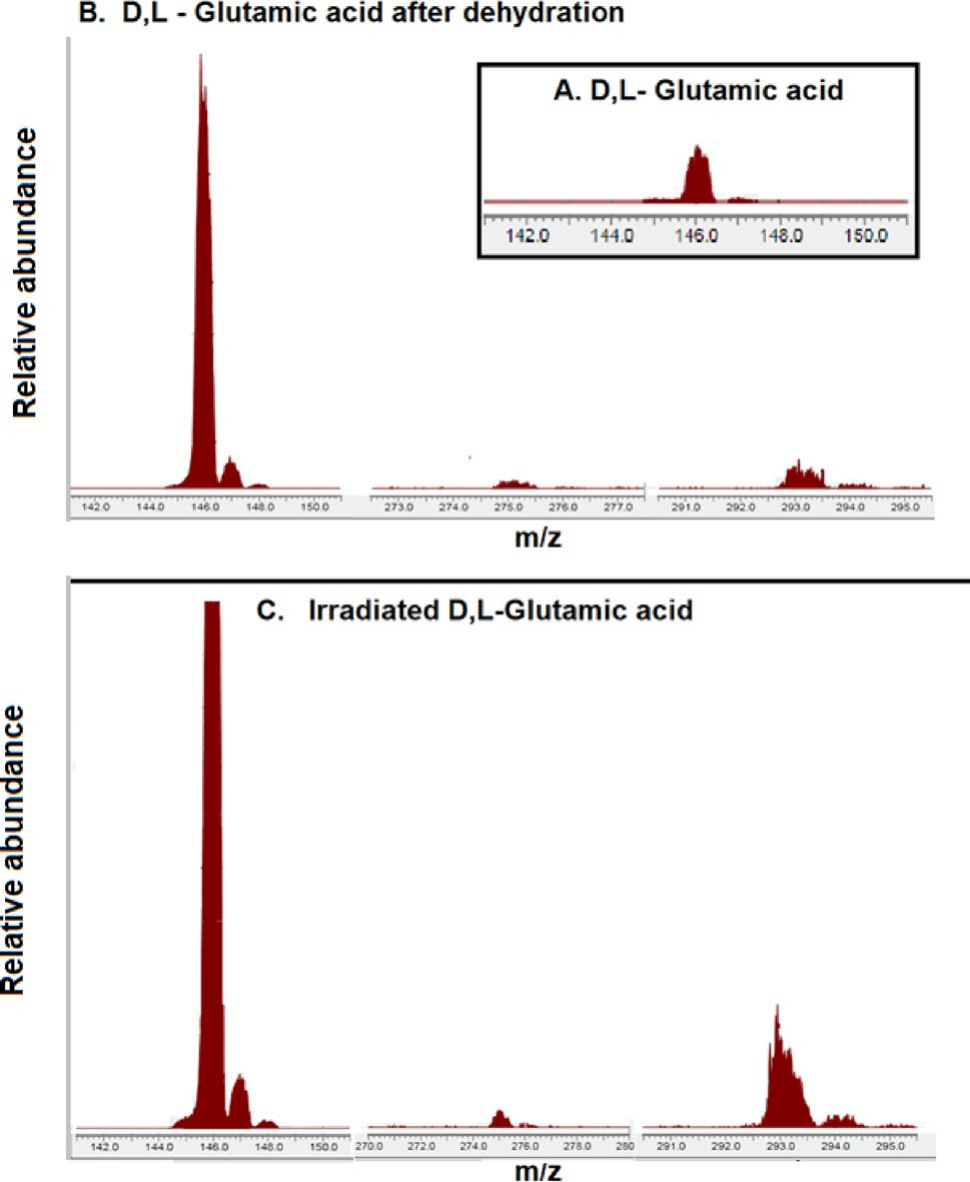
Negative-mode ESI-MS spectra of DL-glutamic acid following 5 wet-dry cycles. **A** Control sample prepared from non-irradiated monomer and subjected to cycling; **B** Experimental sample prepared from pre-irradiated monomer (40 kGy) and subjected to the same cycling process. The peak at m/z 146 corresponds to the monomeric glutamic acid [M - H]^−^. The signal at m/z 275 is assigned to the covalent peptide dimer [2 M - H_2O - H]^−^, which is notably absent or negligible in the control, confirming the role of radiation in amide bond formation. The peak at m/z 293 represents the non-covalent cluster ion [2 M - H]^−^

An electrospray ionization-mass spectroscopy (ESI-MS) detector was used to analyze the m/z of the glutamic acid dimer. A Waters^®^ 515 HPLC pump coupled to a Waters^®^ SQ-2 Single Quadrupole was used. A Mass Detector system, with electrospray ionization in negative (ESI-) mode, was used, adjusting the cone voltage (ca. 20 V) and capillary voltage at 2.55 kV (Waters, Santa Clara, CA, USA). Samples were dissolved in MilliQ water (Fig. 3).

### Attenuated Total Reflectance-Fourier Transform Infrared Spectroscopy (FTIR) Analysis

**Fig. 4:**
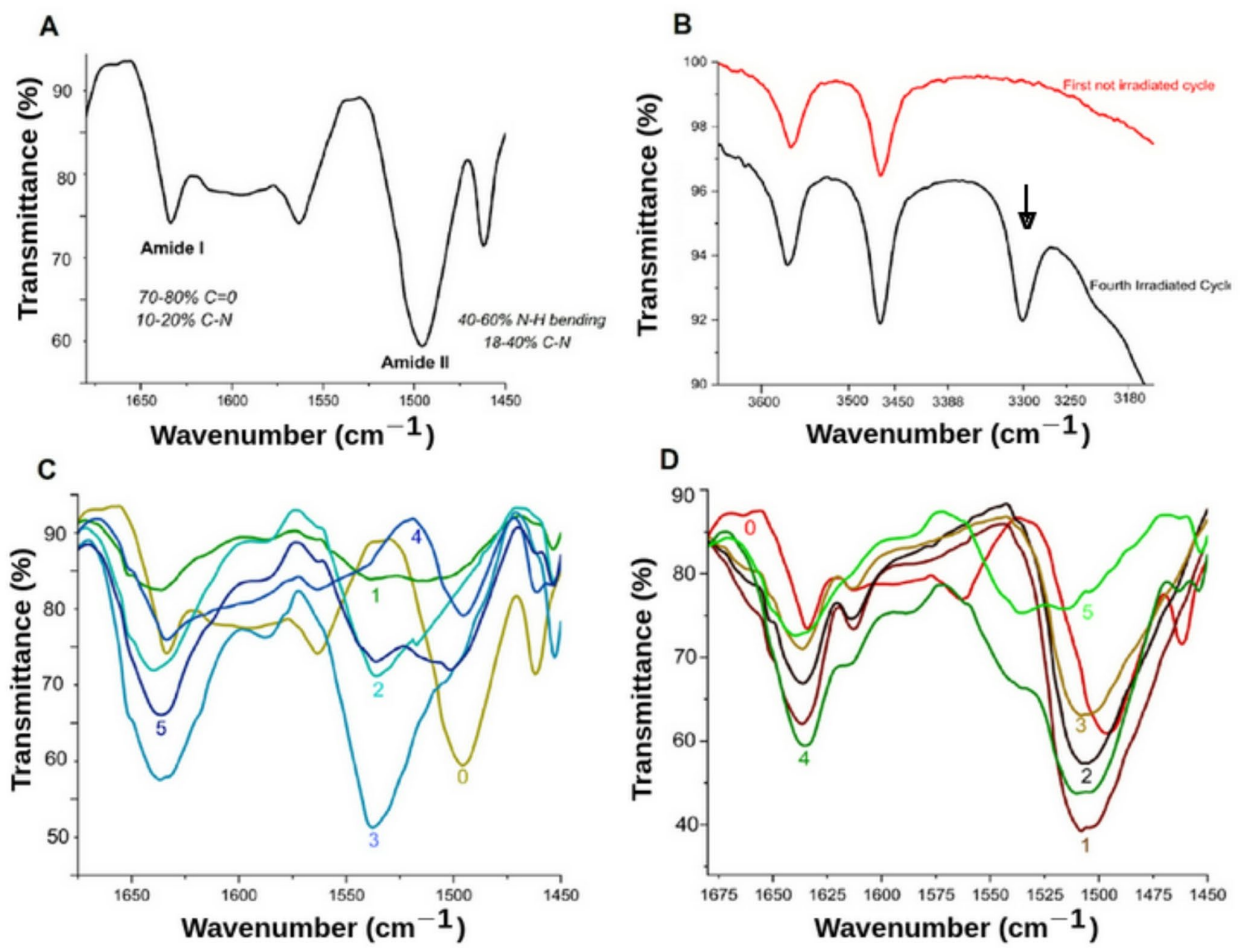
FTIR spectra of DL-glutamic acid evolution. **A** Reference spectrum of the pristine monomer (standard) before any treatment, showing the initial distribution of diagnostic bands. **B** Spectral region near 3300 cm⁻¹ (N–H stretching vibrations), highlighting changes in amine/amide functionalities (black arrow) that support peptide bond formation. **C**, **D** Stacked spectra showing the 1700 –1500 cm⁻¹ region following 5 wet–dry cycles. Numbers (0–5) indicate the cycles in samples prepared with non-irradiated monomer (**C**) and pre-irradiated monomer (**D**). ‘0’ corresponds to the sample after 0 cycles (initial state) for both groups. In **C**, the amide I and II regions display changes in the C = O, C-N (amide I), and N-H, C-N (amide II) bands throughout the five cycles. In contrast, the pre-irradiated samples **D** present the peptide bond region with higher order (sharper bands) and more homogeneous signals. The amide features in **C** and **D** indicate that the fundamental molecular structure remains stable (0) with the further formation of oligomers (1–5) under the wet-dry experimental conditions. The amide I signal at ca. 1650 cm⁻¹ possibly corresponds to a short alpha helix secondary structure. Both **C** and **D** show an increase in molecular weight

Infrared (IR) experiments were conducted using the powder samples with an infrared spectrometer (PerkinElmer Spectrum 100, Norwalk, USA) with an Attenuated Total Reflectance instrument. Measurements were taken with four scans over a wavenumber range from 4000 to 650 cm⁻¹. Hydration and dehydration cycles were also analyzed. Subsequently, the integrals of the curves corresponding to the amide I and II signals in the different cycles were calculated. This approach provides a deeper understanding of how the peptide bond behaves throughout the experimentation. The results of this analysis are presented in Fig. 4.

### Differential Scanning Calorimetry (DSC) and Thermal Gravimetric Analysis (TGA)

Aliquots of 60 µl were drop-cast into 10 aluminum crucibles for Differential Scanning Calorimetry (DSC) and 10 alumina crucibles for Thermal Gravimetric Analysis (TGA). The remaining stock solution was preserved for further analysis. For the subsequent experimental phase, crucible samples were dehydrated in a furnace coupled to a vacuum system at 40 °C (Squaroid Vacuum Oven, Lab-Line Instruments, Illinois, USA) and left overnight. The weighted mass for the samples was 0.5 mg. Analyses were performed using DSC and TGA instruments (Glomro Industrial Co., Ltd., Shanghai, China) at a heating rate of 10 °C/min, starting from approximately 22 °C and reaching a temperature of 400 °C.

Data from each thermogram (DSC and TGA) were analyzed using OriginLab and Qtiplot software to establish a baseline, which was subsequently used to calculate enthalpy values associated with each experiment. Finally, the observed thermal transitions were integrated using the “Integrate” function, and the resulting values were tabulated for subsequent data analysis.

### HyperChem8.0.10 Computer Simulations

Molecular modeling studies were performed using the molecular MM + and semi-empirical *PM3* methods, as implemented in the HyperChem program Version 8.0.10 (HyperCube, Inc., Gainesville, Florida, USA). Geometry optimizations determine molecular structure coordinates corresponding to a potential energy minimum. Geometry optimizations were performed using the HyperChem settings for the MM+ force field, the Polak–Ribiere conjugate gradient algorithm, and a root mean square gradient of 0.0001 kcal/mol. Together in the same workspace, one molecule of D-glutamic acid and one molecule of L-glutamic acid were geometrically optimized to determine the most stable conformation in the “racemic mixture”. After geometry optimization, molecular dynamics (293 K, one ps with a step size of 1 × 10^− 5^ ps) cycles were performed, and energies and dipole moments were obtained for the geometry optimization again. For the *PM3* semi-empirical simulations considering gamma irradiation, the total charge was set to -1, and the spin multiplicity was set to a value different from 1 (to obtain free radicals in the options of the semi-empirical methods menu). The results obtained in HyperChem were computed under gas-phase conditions for the monomers and with a molecule of water for every peptide bond formed. The HyperChem vibrational spectra with active IR vector rendering have been used to display the standard modes associated with the vibrations graphically. The calculation results for the chosen organic molecules are presented. For a precise characterization of the simulated IR bands, a minor horizontal correction was applied to match the well-known amide I and amide II bands.

## Results and Discussion

To evaluate the combined effect of γ-irradiation and wet–dry cycling on DL-glutamic acid, we analyzed structural and thermal changes using multiple complementary techniques. Samples of glutamic acid were either exposed to a single γ-irradiation dose (40 kGy) or kept as non-irradiated controls. Both sets underwent up to five hydration–dehydration cycles at 50 °C. Structural rearrangements and supramolecular organization were examined using polarized light microscopy, while molecular-level changes were assessed through FTIR spectroscopy, and thermal stability was characterized by DSC and TGA analyses. Computational modeling further provided energetic and dipole moment trends across oligomerization steps. The following sections present the results of these combined analyses.

## Polarized Light Microscope

Under the polarized light microscope, the control sample exhibited a mixture of distinct crystalline structures, which depend on the phase of the wet-dry cycle. E.g., before the process of drying, it is possible to observe the formation of tubes, on the other hand, the structures of the dried sample are very different, including well-defined faceted crystals, spheres with complex internal architectures, and elongated rice-like crystals (Fig. 2). These crystals displayed optical polarization activity, confirming their crystalline nature and anisotropic properties. Polarization is a critical indicator of molecular order and structural uniformity, essential for assessing crystal purity and phase behavior. In contrast, the gamma-irradiated samples showed only spherical and rice-like crystals, with no faceted structures observed. All identified crystals in both control and irradiated samples might correspond to the known polymorphs of DL-glutamic acid (Victor et al. 2020). Some authors suggest that those tube-like structures are potential prebiotic chemistry catalysts for molecular evolution (Carny and Gazit 2011).

## Mass Spectrometry

Following a controlled wet-dry cycle, mass spectrometric analysis (Fig. 3) of our electrospun sample reveals a compelling signature: two distinct peaks corresponding to glutamic acid dimers at 293 and 275 m/z. These molecular pairs were formed via: (a) the condensation of two monomeric glutamic acid units, forming a peptidic bond, and (b) forming a covalent bond without the losing of water. This a direct consequence of the dehydration conditions inherent to the cycling process.

Mass spectrometric analysis (Fig. 3) in negative mode identified the peak at m/z \ 275 as the covalent peptide dimer [2Glu - H_2O - H]^−^. The peak at m/z 293 is attributed to a non-covalent cluster ion [2 M - H]^−^, rather than a covalent species. Under the current experimental conditions, neither trimers (m/z 404) nor pyroglutamic acid (m/z 128) were detected above the signal-to-noise threshold. Regarding quantification, the saturation of the monomeric peak m/z 146 and potential differences in ionization efficiencies limit the determination of precise molar yields. However, the consistent emergence of diagnostic amide bands in FTIR complements these findings, suggesting a significant molecular transformation of the bulk sample.

## Attenuated Total Reflectance Fourier Transform Infrared Spectroscopy (FTIR) Analysis

Infrared spectra reveal all the FTIR bands corresponding to DL-glutamic acid (Wancheck and Grasselli 1967). Specifically, for this analysis, the inspection was performed in the distinct transmittance bands corresponding to the amide I and amide II regions. Those regions are sensitive to hydration–dehydration processes (Basyuk et al. 1991; Panuszko et al. 2015; Ervin et al. 2020), providing in detail structural and chemical changes in the samples. Specifically, in samples with no gamma irradiation, well-defined bands are observed in the amide I (~ 1700–1600 cm⁻¹) and amide II (~ 1600–1550 cm⁻¹) regions, characteristic of C = O and N–H bond vibrations, respectively (Bellamy 1975; Kong and Yu 2007a; Jabs). These documented spectra indicate that the fundamental molecular structure of glutamic acid remains stable under the initial experimental conditions. In contrast, the irradiated samples exhibit significant spectral changes in these key regions (Fig. 4). All the ATR-FTIR spectra corresponding to amide or peptide bond signals were explored in more detail (Fig. 4). The analysis of amide bands provides insight into amino acid oligomerization (Fig. 4A). In addition to the general features of the amide I/II regions, the FTIR spectra revealed distinct bands at ~ 1652 and ~ 1636 cm⁻¹. These signals fall within the canonical range of the amide I vibration, which is highly sensitive to hydrogen bonding and local conformational order (Byler and Susi 1986; Barth 2007). Specifically, the band at ~ 1652 cm⁻¹ is consistent with α-helical conformations, while the component near ~ 1636 cm⁻¹ is characteristic of β-sheet structures. The emergence of these features suggests that, beyond simple oligomerization, partial ordering into secondary motifs may occur under the experimental conditions employed. The observation of α-helix- and β-sheet–like signatures in the amide I region is noteworthy, as such conformational preferences may reflect early stages of structural organization relevant to prebiotic peptide evolution (Krimm and Bandekar 1986; Kong and Yu 2007b; Barth 2007). A more detailed inspection of the intensities and shifting of peptide bond bands is central to understanding the change in molecular mass and secondary structure formation. Our study observed peptide bond bands at approximately 1634 cm⁻¹ for the amide I band and around 1510 cm⁻¹ for the amide II band (Fig. 4A-C). These two bands are shown in Fig. 4. Notably, in the gamma irradiated samples, the intensities of the amide I and II bands decrease markedly. New infrared features emerge, which are potentially associated with the formation of peptide bonds (N-H band from peptide bond at 3300 cm⁻¹ in Fig. 4B). This behavior suggests that ionizing radiation induces cleavage of original bonds followed by molecular reorganization and oligomerization (Fig. 4D). Moreover, the O–H stretching region (~ 3400 cm⁻¹) exhibits a notable band broadening, indicative of diversification of the enhanced hydrogen bonding interactions, which decrease the molecular order and possibly result from the development of more complex molecular networks. Moreover, authors have found that the amide I band tends to be larger than the amide II band, thereby revealing the associated secondary structures (Murphy et al. 2014; Sadat and Joye 2020; De Meutter and Goormaghtigh 2021; Chatterley et al. 2022). A notable change in the spectra is the alteration in the symmetry of the amide I and II bands, with the amide II band being significantly larger than the amide I band (Fig. 4). Additionally, the amide II band is observed at 1510 cm^− 1^ (not at ca. 1550 cm^− 1^). This could be correlated with the formation of the secondary structure (Ross and Deamer 2016). The location of the detailed amide bands is crucial for determining secondary structures (Ye et al. 2024). Additional infrared signals between 3600 –3400 cm^− 1^ correspond to -OH bonds, and in the gamma irradiated spectrum, the new N-H band (3300 cm^− 1^) suggests the formation of the peptide bond (Buontempo et al. 1972). The “fingerprint region” (~ 1500 –700 cm⁻¹, not shown) also displays marked differences between control and irradiated samples. These variations reflect specific changes in the molecular conformation of glutamic acid, potentially associated with the formation of novel compounds or the fragmentation of the original molecular framework.

The evolution of the Amide I and II regions (1500–1700 cm⁻¹) during the wet-dry cycles reveals a distinct path toward molecular complexity. In the non-irradiated control (Fig. 4C), the fluctuations in peak intensity suggest a stochastic formation and reorganization of disordered oligomers. In contrast, the pre-irradiated samples (Fig. 4D) exhibit a trend toward spectral uniformity and bandwidth narrowing. Although the absolute intensity of these bands is lower than in the control, their sharpness indicates a higher degree of conformational order and the formation of a more homogeneous population of peptide-linked structures.

**Fig. 5:**
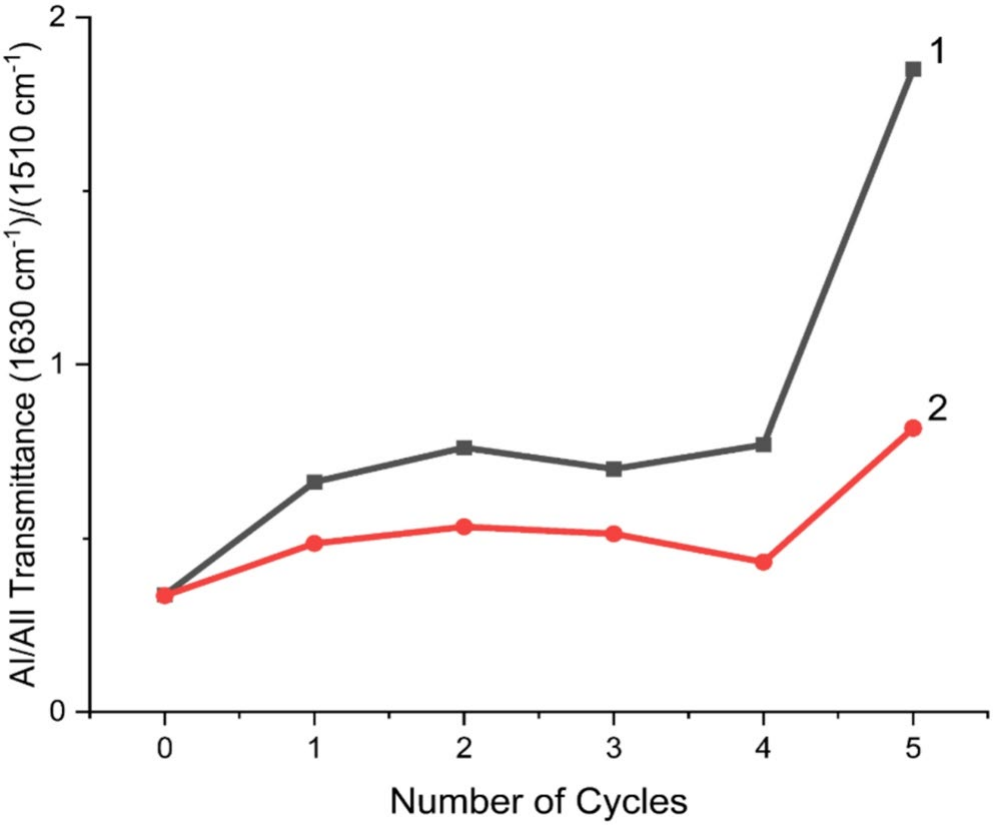
The behavior of the amide I/amide II intensity ratio through the different wet-dry cycles is presented. The control samples (1) exhibit a moderate increase up to cycle 4, at which point, a difference between the values is observed. Overall, the trend shows a gradual increase in the ratio values, indicating the formation of peptide bonds in control (1) and gamma-irradiated samples (2). The gamma-irradiated samples exhibit similar upward behavior values. A pronounced increase is observed in the final cycle

This behavior suggests that gamma radiation acts as a ‘structural primer,’ likely creating radical-induced nucleation sites in the solid-state monomer that guide the subsequent thermal polymerization during wet-dry cycles. The emergence of a signal at approximately 1650 cm⁻¹ is particularly noteworthy, as it suggests the stabilization of short alpha-helix-like secondary structures, which are typically more resistant to further disordered growth but contribute to the overall increase in molecular weight and thermal stability observed in the DSC and MS analyses (Barth 2007; Mamajanov et al. 2014). Furthermore, the wavenumber positions of the amide bands for the controls and irradiated samples were consistent. In a subsequent analysis to detail the change in the bands related to chemical composition, the ratio of Amide I/Amide II C = O/(N-C, N-H) of the peptide was obtained (Fig. 5). For a more detailed data analysis, it was decided to apply a ratio to these two bands, allowing observation of both the behavior of the C = O and the C-N bonds.

The trifunctional structure of DL-glutamic acid allows for multiple bonding pathways, potentially yielding a mixture of alpha-peptides and gamma-peptides. Our analytical approach focused on the diagnostic signatures of the peptide bond. The ESI-MS results confirmed the synthesis of dimers (293 and 275 m/z), while FTIR analysis identified distinct Amide I and II bands consistent with the formation of a-helix-like (~ 1652 cm⁻¹) and b-sheet (~ 1636 cm⁻¹) configurations. Although these findings demonstrate the successful promotion of peptide bond formation through the synergy of ionizing radiation and wet-dry cycles, the precise isomeric distribution of these products remains to be characterized in future studies using regioselective separation techniques (Table 1).

**Table 1:** Values obtained from the integrals of Amide I (C = O) and Amide II (N-C, N-H)

Control Samples						Gamma Irradiated Samples					
Number of cycles	Amide I (area)	Amide II (area)	Quotient AI/AII	Amide I band [cm^− 1^]	Amide II band [cm^− 1^]	Number of cycles	Amide I area	Amide II area	Quotient AI/AII	Amide I band [cm^− 1^]	Amide II band [cm^− 1^]
0	224.00	664	0.34	1634	1500	0	195.00	582	0.34	1635	1500
1	295.00	445	0.66	1637	1506	1	535.00	1102	0.49	1636	1509
2	508.00	667	0.76	1640	1507	2	445.00	834	0.53	1636	1508
3	882.00	1260	0.70	1637	1507	3	368.00	717	0.51	1637	1509
4	603.00	228	2.64	1635	1508	4	528.00	1222	0.43	1635	1509
5	783.00	423	1.85	1636	1515	5	402.00	492	0.82	1637	1523

## Differential Scanning Calorimetry (DSC) and Thermal Gravimetric Analysis (TGA)

**Fig. 6:**
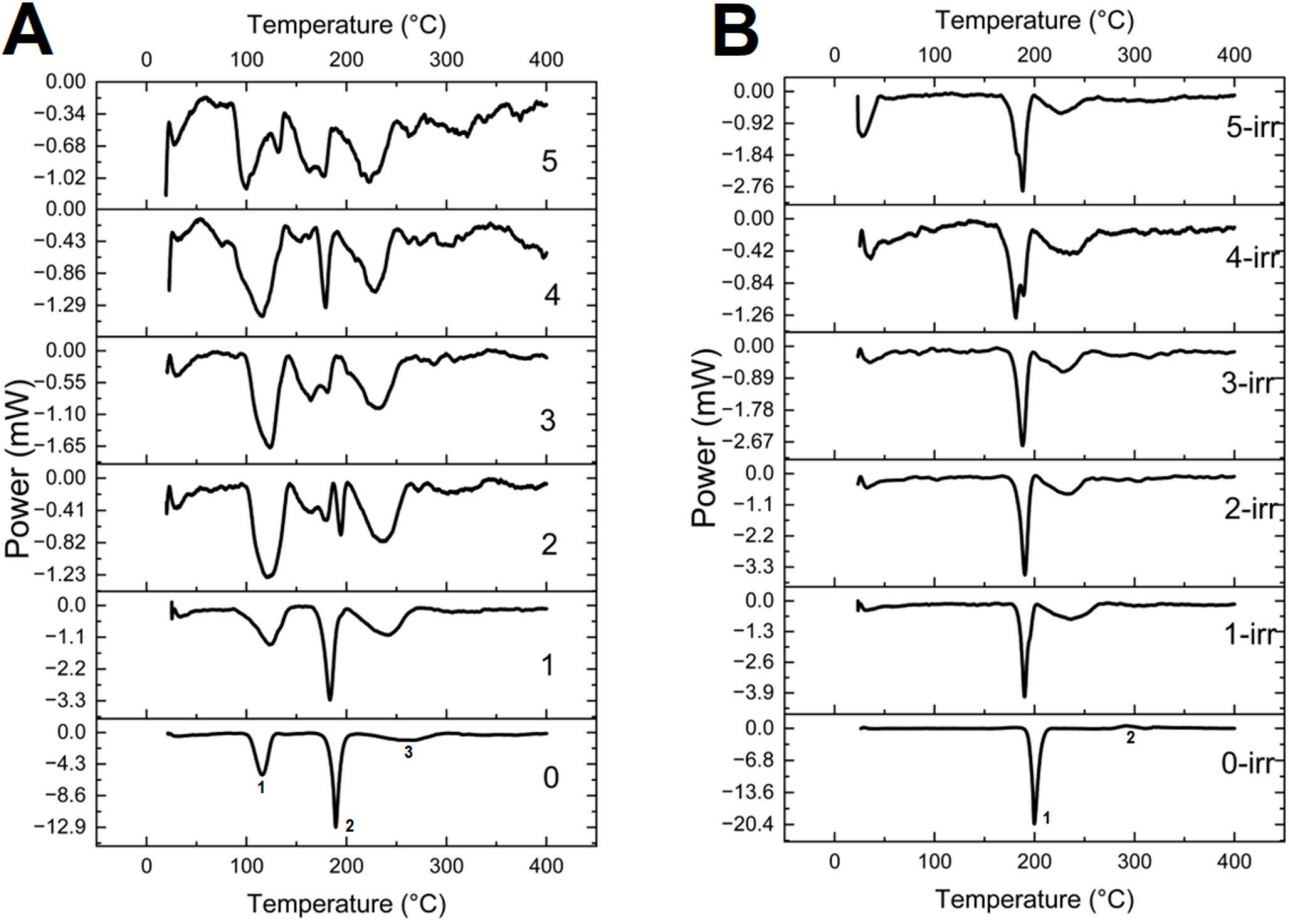
DSC thermograms of the **A** DL-Glutamic acid without gamma irradiation and (B) the gamma-irradiated ones. Control in **A** exhibits three noticeable endotherms at 116 °C (1), 189 °C (2), and 261 °C (3), while the gamma irradiated **B** ones exhibit two at 190 °C (1) and 235 °C (2). This change in thermal transitions can be attributed to the loss of hydrogen from the alpha carbon of the amino acid (Spinks and Woods 1990). First thermograms in A and B (“control” and “irradiated Sample”) were not self-assembled through the wet-dry cycles

For Differential Scanning Calorimetry (DSC) and Thermal Gravimetric Analysis (TGA), a collection of thermograms is obtained in two sets (1) non-irradiated samples and (2) gamma-irradiated samples, through five wet-dry cycles (Fig. 6). The non irradiated samples before the wet-dry cycles exhibit three noticeable thermal transitions (1–3 thermal transitions in Fig, 5 A at 116 °C, 189 °C, and 261 °C). In contrast, the gamma-irradiated ones exhibit two (1,2 thermal transitions in Fig. 5B at 190 °C and 235 °C).

In this case, the enthalpy values associated with the thermal transition varied significantly, with the controls and the upcoming wet-dry cycles showing a notable progressive decrease in enthalpy values. This result may indicate a change in the thermal stability from samples without gamma irradiation (e.g., 261 °C), contrasting with the irradiated samples (235 °C), as seen previously (Cataldo et al. 2011). The wet-dry cycles result in thermal transitions that gradually increase the thermal stability (fewer thermal transitions in thermograms). The interaction with different molecular environments results in these thermal changes (Heredia et al. 2013). An example of this increase in thermal stability is seen during the first hydration–dehydration cycle, where a signal is observed in controls (Fig. 6A, graph 1) at 115 °C, which disappears in the first cycle and subsequent results. These shifts may indicate the formation of the peptide bond. Figure 6B shows a general increase in stability but with fewer thermal transitions, possibly due to the higher molecular interactions (tighter interactions) when the alpha carbon has a free radical.

**Fig. 7:**
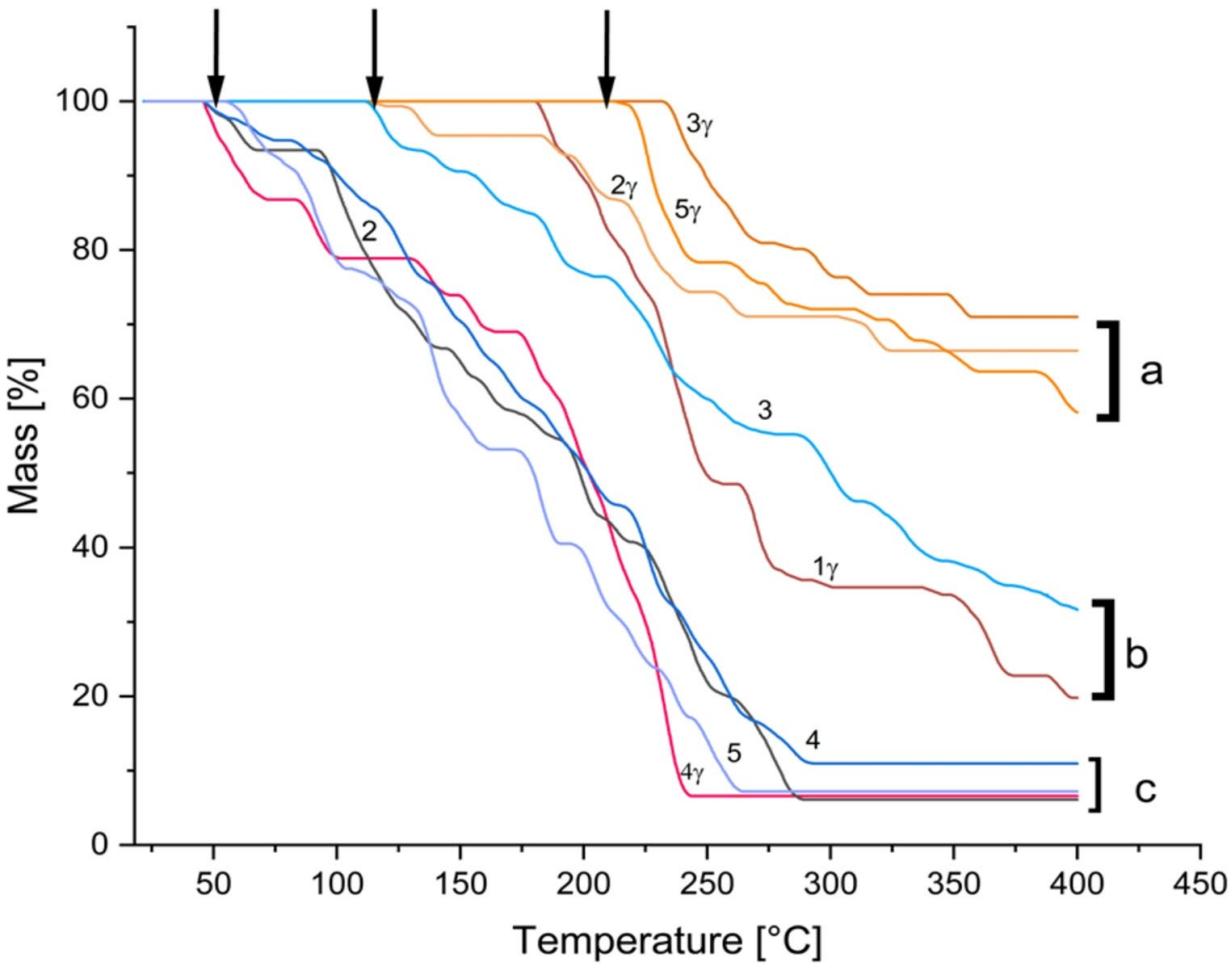
TGA thermograms of DL-glutamic acid without and with gamma irradiation (). Numbers represent the wet and dry cycles performed on the sample

To understand the phenomenon underlying the DSC thermal transitions in depth, Thermogravimetric analysis was performed. After the wet-dry processes, different TGA thermograms were obtained (Fig. 7). The samples did not exhibit changes in mass until ~ 50 °C (Fig. 7, first arrow from left to right). Changes were observed in samples with no irradiation (graphs 2, 4, and 5 in Fig. 7) and in the gamma-irradiated samples (graph 4γ, Fig. 7). Another characteristic of these results is the ~ 7% mass loss. Moreover, graphs 1γ and 3 indicate that the thermal transition initiates at approximately 112 °C. The corresponding thermogram of sample 1γ has a similar onset of thermal transition, however, with a different mass loss. This increase in stability is seen in the change in the onset of the thermal transition from approximately 50 °C to 112 °C. This result might indicate a change in the molecular packing in the crystal structure. The gamma irradiated samples named “group a” (Fig. 7, Graphs 2γ,3γ,5γ) have thermal transitions starting at 180 °C, 213 °C, and 232 °C, respectively. Furthermore, although the starting temperature of decomposition for sample 2γ is 115 °C, it preserves the mass at a higher rate of 67%. The other two samples, 5γ and 3γ, began to lose weight at 212 °C and 230 °C, respectively. They preserve their mass to 58% for the 5γ sample and 70% for the 3γ sample.

## Computational Analysis

**Fig. 8:**
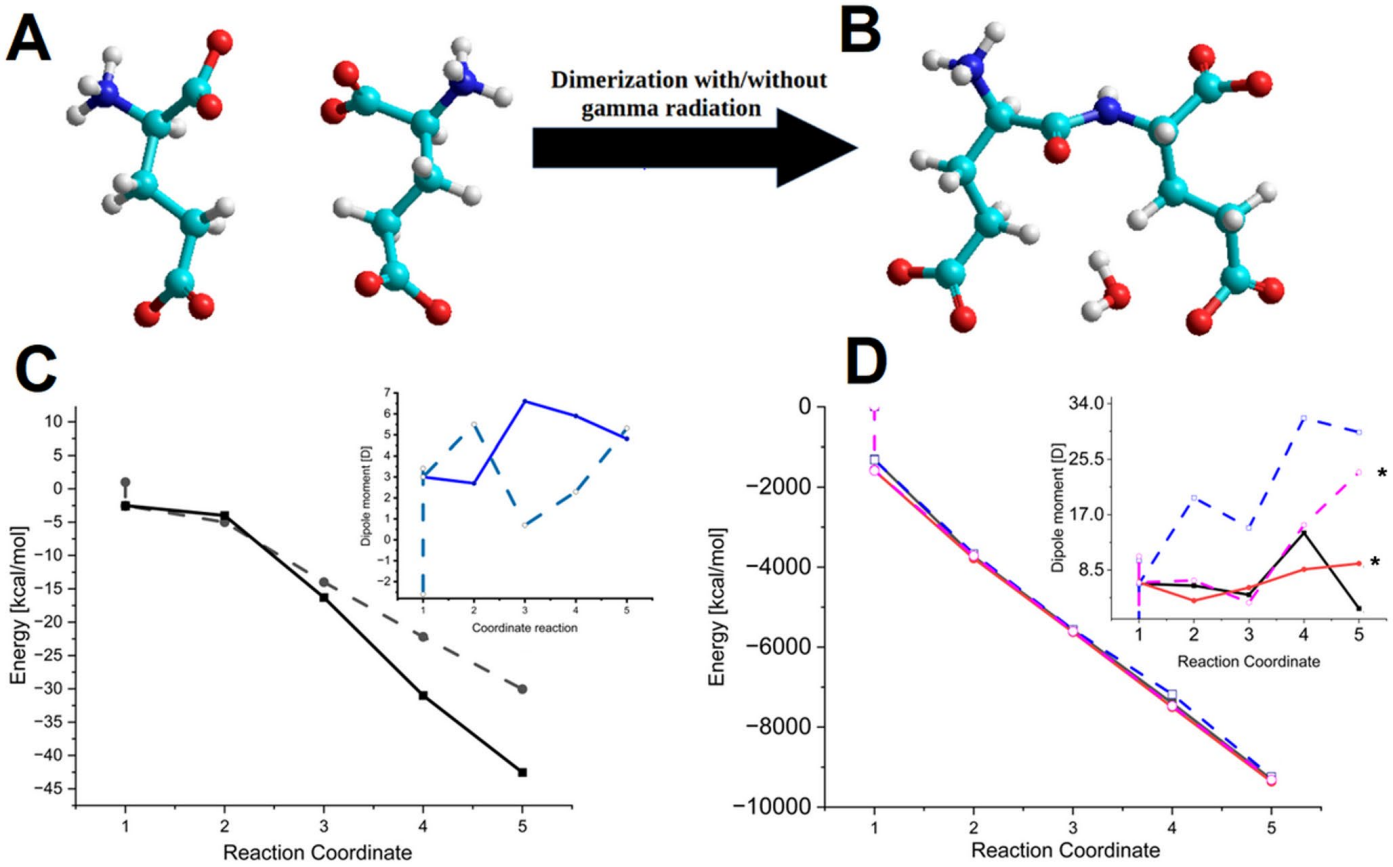
Computational analysis of glutamic acid dimerization. In **A**, the D and L-glutamic acid monomers, and in **B**, the D, L-diglutamic acid dimer. MM+ (**C**) and *PM3* (**D**) molecular modeling of the potential energies with the change in dipole moment values (insets) for both separated (dashed lines) and bonded amino acids (solid lines). Values marked with (*) represent the free radical added to the *PM3* simulation

MM+ molecular mechanics and *PM3* semi-empirical simulations were performed to gain insight into the possible interactions between chemical groups from individual D- and L-glutamic acid and the racemic mixtures, facilitating the assembly and further oligomerization to form larger complexes. In Fig. 8 (A and B), a dimerization shows the most favorable conformation (Fig. 8A and B). Variations in potential energy values and dipole moments (Fig. 8C and D and insets) arise from the use of the different MM+ molecular mechanics and *PM3* semi-empirical theoretical levels. Fig. A and B depict the simulation of the racemic dimerization process. In 8C, MM+ potential energy and dipole moment values for separated (dashed line) glutamic acid molecules and the bonded (solid line) D- and L-amino acids. In D, PM3 semi-empirical potential energies and dipole moment values (inset) for separated (dashed line) and bonded (solid line) D- and L-amino acids.

Figure 8C and D present the energy and dipole moment (inset) values obtained at the MM + and *PM3* levels of theory. Continuous lines correspond to amino acids covalently linked through peptide bonds (oligomers), while dashed lines represent the same number of amino acids in their free, monomer state. Figure 8C and D correspond to the energy profiles, displaying generally decreasing values towards a more stable conformation. In the same sense, curves in the insets depict the dipole moment, showing an overall increase. In MM+, amino acids that have undergone covalent linkage exhibit slightly lower total energies compared to their free counterparts. The behavior of the dipole moments yields higher values than their initial counterparts, possibly associated with chemical reactivity.

Figure 8D displays data obtained using the *PM3* level of theory. Curves with an asterisk (*) represent values with free radicals that display fluctuating values. Overall, these results indicate that increasing the number of amino acids leads to a decrease in total energy and a general increase in dipole moment. Regardless of the configuration, the *PM3* energy follows an almost linear behavior, decreasing to a final value of approximately − 5600 kcal/mol, as shown in Fig. 7D. Finally, Fig. 8C and D (inset) show increasing values for the dipole moment across various structural configurations (Table 2).

**Table 2:** MM + and PM3 computer simulation values of the oligomerization process of chiral-dependent glutamic acid under wet-dry conditions without and with free radicals (“gamma radiation”)

Simulation of D and L amino acids*/**	MM+			PM3		PM3 free radical (*)	
	Number of amino acids	E [kcal/mol]	Dipolar moment (D)	E (kcal/mol)	Dipolar moment (D)	E (kcal/mol)	Dipolar moment (D)
D	1	-2.6	3.4	-1301	9.9	-1566	10.6
L	1	-2.5	3	-1315	6.4	-1588	6.6
D, L	2	-5	5.5	-3666	19.6	-3716	6.9
D-LH20	2	-4	2.7	-3738	6.1	-3783	3.8
D, L,D	3	-14	0.7	-5564	15	-5604	3.5
D-L-D 2(H20)	3	-16.3	6.6	-5574	4.7	-5624	5.8
D, L,D, L	4	-22.2	2.3	-7180	31.8	-7470	15.41
D-L-D-L 3(H20)	4	-31	5.9	-7401	14.2	-7500	8.6
D, L,D, L,D	5	-30.04	5.31	-9242	29.6	-9316	23.51
D-L-D-L-D 4(H20)	5	-42.55	4.81	-9304	2.6	-9362	9.5

^*Comma−separated simulations are amino acids without a peptide bond^

^**Hyphens in simulations represent amino acids with a peptide bond^

These results show that the non-irradiated and gamma-irradiated samples of glutamic acid increase their molecular weight throughout wet-dry processes. This fact suggests that the molecular evolution routes were present in a diversity of conditions in primitive Earth, thus guaranteeing the physicochemical dynamics towards prebiotic complexity independently of the presence of gamma radiation. This is supported by polarized light images and infrared spectroscopy, where irradiated samples exhibit a more complex behavior and more homogeneous amide I and II signals compared to non-irradiated samples (Figs. 2, 3, 4 and 5). This observation implies that while both irradiated and control samples undergo molecular weight increase due to wet-dry cycling, subtle structural differences arise, likely influenced by irradiation-induced modifications. Mass spectrometry confirms the increase in the molecular weight (Fig. 3A and B). A notable shift of the amide II band to lower wavenumbers in gamma-irradiated crystals suggests a stronger hydrogen-bonding network, possibly due to a more compact crystalline arrangement where hydrogen atoms from α-carbons are absent. This structural hardening is further corroborated by thermal analysis, which reveals that gamma-irradiated samples exhibit higher stability than non-irradiated samples, as evidenced by thermal transitions shifting to higher temperatures except for the thermal transition at 261 °C in non-irradiated samples. Interestingly, wet-dry cycling enhances thermal stability in both non-irradiated and irradiated samples, with successive cycles progressively reducing the number of endothermic transitions. By the fifth cycle, only minimal thermal changes are observed, indicating that repeated hydration-dehydration events drive the system toward more stable oligomeric states. This finding aligns with computer simulations, which predict not only increased stability but also elevated dipole moments in these oligomers, suggesting a possible physicochemical activity. The latter suggests a potential rise in catalytic activity, which might have significant implications for prebiotic chemistry and the emergence of proto-metabolic processes. Overall, these results highlight how environmental factors, such as gamma radiation and cyclic hydration-dehydration, can shape peptide stability and possibly reactivity, thus facilitating molecular evolution processes in prebiotic scenarios. Computational analysis in this study, performed using MM+ molecular mechanics and *PM3* semi-empirical methods, provided key structural, energetic, and vibrational insights that complement the experimental findings and reveal possible mechanisms not directly accessible through spectroscopy or thermal analysis alone. Energy minimization of monomers, dimers, trimers, tetramers, and pentamers showed a consistent decrease in total energy with increasing chain length, indicating that aggregation and oligomerization are thermodynamically favorable under the modeled conditions. Simulations incorporating radical species—mimicking the primary products of γ-irradiation—revealed slightly altered energies and dipole moments compared to their neutral counterparts, suggesting radical–radical recombinations.

## Conclusions

In conclusion, our integrated analytical approach demonstrates that the synergistic application of gamma radiation (40 kGy) and wet-dry cycling effectively promotes peptide bond formation and oligomerization in DL-glutamic acid under conditions simulating the prebiotic environment. This was conclusively verified across multiple scales of analysis. Polarized light microscopy revealed a fundamental radiation-induced morphological transformation, from simple needle-like crystals to complex spherical aggregates with birefringent cores, indicating significant supramolecular reorganization. Mass spectrometry provided direct evidence of covalent bonding, confirming the formation of the peptide dimer at m/z 275. This species is clearly distinguishable from the non-covalent cluster observed at m/z 293, which lacks the dehydration characteristic of a true peptide bond. Critically, Fourier-transform infrared spectroscopy analysis suggests a delineated structural evolution pathway, indicating a transition from alpha-helix-like motifs in control samples towards beta-sheet conformations in the irradiated oligomers, a shift marked by attenuated amide I (1636 cm⁻¹) and enhanced amide II (1510 cm⁻¹) bands alongside a new N-H stretch (3300 cm⁻¹). Thermal analyses robustly supported the formation of more stable molecular architectures. Differential Scanning Calorimetry showed a complete suppression of the original thermal transition (116 °C) and the emergence of a new, higher-temperature endotherm (190 °C), with decreasing enthalpy values pointing towards increased stability. Thermogravimetric analysis corroborated this by showing reduced structural water retention in irradiated samples, consistent with a denser, more compressed oligomeric structure, possibly a product of the free radical formation after gamma irradiation. Computational modeling further affirmed the enhanced stability and formation of these samples without gamma radiation and radiation-synthesized oligomers. Collectively, these findings establish a compelling model in which prebiotic factors—ionizing radiation and cyclic hydration-dehydration—act in concert to overcome kinetic and thermodynamic barriers to polymerization of the racemic glutamic acid. This study, utilizing a racemic mixture, provides a critical foundation for future investigations into chirality effects and the potential catalytic properties of these primordial oligomers on other low molecular weight compounds. Elucidating the complete mechanistic pathway of this prebiotic oligomerization and its functional outcomes remains a primary objective for understanding plausible routes of prebiotic molecular evolution on early Earth and beyond.
